# The impact of curcumin on livestock and poultry animal's performance and management of insect pests

**DOI:** 10.3389/fvets.2023.1048067

**Published:** 2023-02-03

**Authors:** Anjana Sureshbabu, Elena Smirnova, Adhimoolam Karthikeyan, Mohammad Moniruzzaman, Senthil Kalaiselvi, Kiwoong Nam, Gaelle Le Goff, Taesun Min

**Affiliations:** ^1^Department of Animal Biotechnology, Jeju International Animal Research Center (JIA) and Sustainable Agriculture Research Institute (SARI), Jeju National University, Jeju, Republic of Korea; ^2^Subtropical Horticulture Research Institute, Jeju National University, Jeju, Republic of Korea; ^3^Department of Biochemistry, Biotechnology and Bioinformatics, Avinashilingam Institute for Home Science and Higher Education for Women, Coimbatore, Tamil Nadu, India; ^4^DGIMI, Univ Montpellier, INRAE, Montpellier, France; ^5^Université Côte d'Azur, INRAE, CNRS, ISA, Sophia Antipolis, France

**Keywords:** curcumin, feed additive, insects, plant-based natural products, livestock and poultry animals

## Abstract

Plant-based natural products are alternative to antibiotics that can be employed as growth promoters in livestock and poultry production and attractive alternatives to synthetic chemical insecticides for insect pest management. Curcumin is a natural polyphenol compound from the rhizomes of turmeric (*Curcuma spp*.) and has been suggested to have a number of therapeutic benefits in the treatment of human diseases. It is also credited for its nutritional and pesticide properties improving livestock and poultry production performances and controlling insect pests. Recent studies reported that curcumin is an excellent feed additive contributing to poultry and livestock animal growth and disease resistance. Also, they detailed the curcumin's growth-inhibiting and insecticidal activity for reducing agricultural insect pests and insect vector-borne human diseases. This review aims to highlight the role of curcumin in increasing the growth and development of poultry and livestock animals and in controlling insect pests. We also discuss the challenges and knowledge gaps concerning curcumin use and commercialization as a feed additive and insect repellent.

## 1. Introduction

Development of animal performance without disease is great prominence in increasing livestock and poultry production. Relying on antibiotics to enhance the quantity and quality of meat of food animals has been widely depended upon for a long time. However, scientists claim that conflict over antibiotic residues and resistance have also emerged as an outcome of the overuse of antibiotics (1, 2). In this context, finding alternatives to antibiotics to improve animal performance by promoting gut health has gained interest. Using plant-derived natural products and their analogs instead of antibiotics for improving animal performance and wellbeing are widely accepted eco-friendly strategies. It is also an effective alternative to synthetic chemical insecticides for insect pest management. Previously, plant-derived natural products were used in animal nutrition for their numerous beneficial effects such as antimicrobial activity, ability to promote gut health of the animals, flavoring agents in feed due to their aromatic value, etc. (1, 2). A humongous number of research reveals the efficacy of plant products, especially the productive reuse of “waste” parts such as citrus peels as feed for livestock, which can also be considered a strategy to recycle the peels (3). Another approach is to isolate the natural substances from plants and their by-products used as traditional medicines in the past (4), including flavonoids, polyphenols, anthocyanins, etc. (1, 5, 6).

Curcumin, also known as diferuloylmethane (C_21_H_20_O_6_), is a hydrophobic polyphenolic phytocompound present in the rhizomes of the turmeric (*Curcuma. spp*) belonging to the family of Zingiberaceae which is commonly found in Asian countries. It is the major constituent of turmeric powder, widely used as a culinary spice and traditional drug. Turmeric isolated from the rhizomes of the plant *C. longa*, has also been very well-known for its medicinal benefits for decades. Curcumin is the active ingredient of turmeric that owes its yellow color (4). Turmeric consists of 60–70% carbohydrates, 6–8% proteins, 5–10% fat, 3–7% minerals, and 6–13% moisture (7). Although, 3–5% of curcuminoids, include more than 50 structurally related compounds. Three main compounds include, curcumin, demethoxycurcumin, and bisdemethoxycurcumin (8). Curcumin is biosynthesized from two molecules of feruloyl-CoA and one molecule of malonyl-CoA *via* two enzymatic conversions, catalyzed by DIKETIDE-CoA SYNTHASE (DCS) and CURCUMIN SYNTHASE (CURS). Both DCS and CURS belong to the type III polyketide synthase family (9–11). Curcumin is reported to have effects as an antioxidant and anti-inflammatory agent (12–14). As a consequence, it has been used to treat oxidative and inflammatory disorders, metabolic syndrome, arthritis, anxiety, hyperlipidemia, cancers (i.e., Lung cancer, bladder cancer, and breast cancer), and neurological disorders (12, 15–20). Curcumin also possesses nutritional and insecticide properties improving poultry and livestock animal's production performances and broad-spectrum activity against insects that damage the agricultural crops and can transfer diseases to human.

Curcumin is recently being increasingly preferred by animal nutritionists as an alternative to chemical additives such as chemotherapeutic drugs and antibiotics in animal feed because animal production industries are under pressure to improve the animal production performances, decrease the economic losses, and confirming the safety of products for human consumption (21, 22). The usage of curcumin has made remarkable advancements in a wide range of nutritional aspects across growth, reproductive capacity, digestibility, stress response, immune functions, and histopathology in different age stages of monogastric animals such as pigs, poultry, and fish (23). Curcumin supplementation reduces absolute and abdominal fat weights by regulating lipid metabolism in broiler chickens (24). The dietary addition of curcumin improve meat quality, alleviate oxidative stress, and reduce fat deposition in pigs as well (25). Curcumin also owns broad-spectrum activity against insect pests. Arthropod vectors are responsible for driving and spreading the diseases such as malaria, dengue, chikungunya to human (26) and also for causing significant damage to agricultural crops (27). The climate changes may have impact on insect mutation (25, 26). But, the prevalent use of chemical insecticides on large populations of insects over space and time quickly demonstrated the direct relevance of the mutation process to insect control with the rapid development of insecticide resistance. Also, Insects very quickly evolved heritable, stable, qualitative or quantitative changes in their genomes that rendered many chemicals largely ineffective. In this context, the environment friendly approach to insect management include the use of natural compound based botanical insecticides (28). The bioactive compounds in the form of essential oil from turmeric extracts have insecticidal properties, with curcumin being the most active chemical and acting as a natural insecticide (29). The larvicidal activity of curcumin make them appropriate environment friendly vector control agent (30). Curcumin was reported to induce autophagic cell death in *Spodoptera frugiperda* cells *in vitro*, reported the first cytotoxic effect of curcumin on insect cells (31). Similarly, curcumin and its derivatives (demethoxycurcumin, curcumin-BF2 complex, and a monocarbonyl tetramethoxy curcumin) exhibited larvicidal activities in *Culex pipiens* and *Aedes albopictus* mosquitoes vectors even though no specific structure-activity relationship was clear enough to describe the effect of curcumin (32).

Despite the overwhelming therapeutic research on curcumin, little work has been done to describe curcumin's role in improving poultry and livestock production and controlling insect pests. This review mainly discusses how curcumin plays a role in improving the life quality of poultry and livestock animals and managing insect pests. We also mention challenges and research gaps related to curcumin use and issues concerning commercialization as a feed additive and insect repellent.

## 2. Chemical structure and various benefits of curcumin

**Figure 1:**
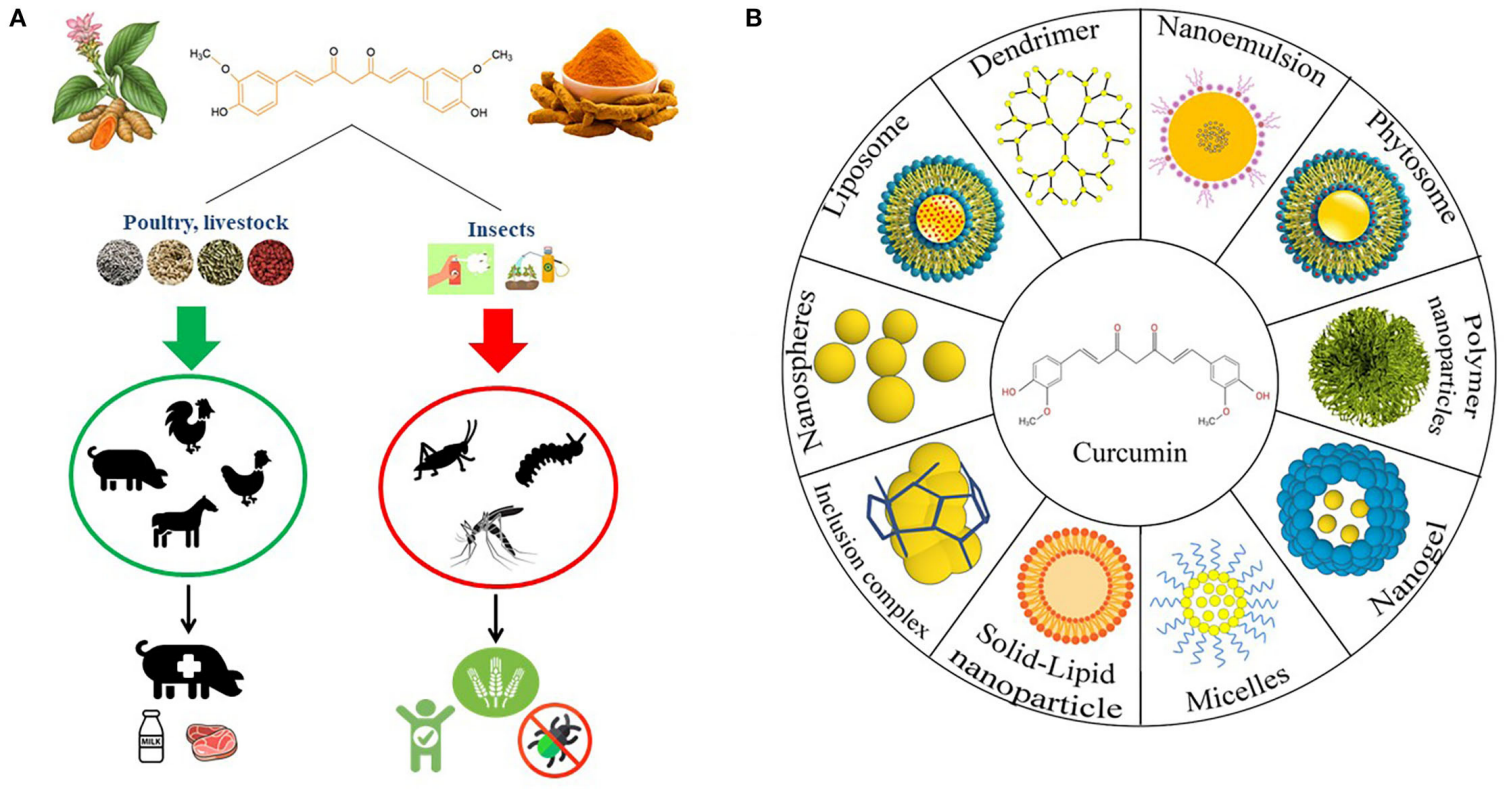
**(A)** Curcumin effects on livestock, poultry and insects when supplemented or used as a feed additive and insecticides. **(B)** The different types of curcumin nanoformulations.

Curcumin is a symmetrical molecule with IUPAC name (1E,6E)-1,7-bis (4-hydroxy-3-methoxyphenyl)-1,6-heptadiene-3,5-dione and a molecular weight of 368.38 (33). Curcumin has a structure with two phenols and potentially enolizable β-diketone moieties, which are conjugated by two allylic double bonds (34). Curcumin exists mostly in a hydrogen-bond-stabilized keto-enol state. In polar solvents, curcumin exists in diketo form, and in non-polar solvents, it exists in the enol form. In basic media, the enol form of curcumin dominates and acts as an electron donor, whereas in neutral and acidic media, the keto form of curcumin is dominated and acts as a proton donor. Curcumin is soluble in dichloromethane, chloroform, dimethyl sulfoxide, acetone, and ethyl acetate, but it is insoluble in water and other diethyl ethers. Curcumin possesses light sensitivity and is unstable in alkaline solutions (8). Along with curcumin, other naturally occurring curcuminoids, such as demethoxycurcumin (15%) and bisdemethoxycurcumin (5%), are also biologically active turmeric constituents with significant health benefits (35, 36). While curcumin is naturally derived, its derivatives are generally produced by a chemical reaction between aryl-aldehydes and acetylacetone (37). Benefits of using curcumin are summarized in Figure 1.

**Figure 2:**
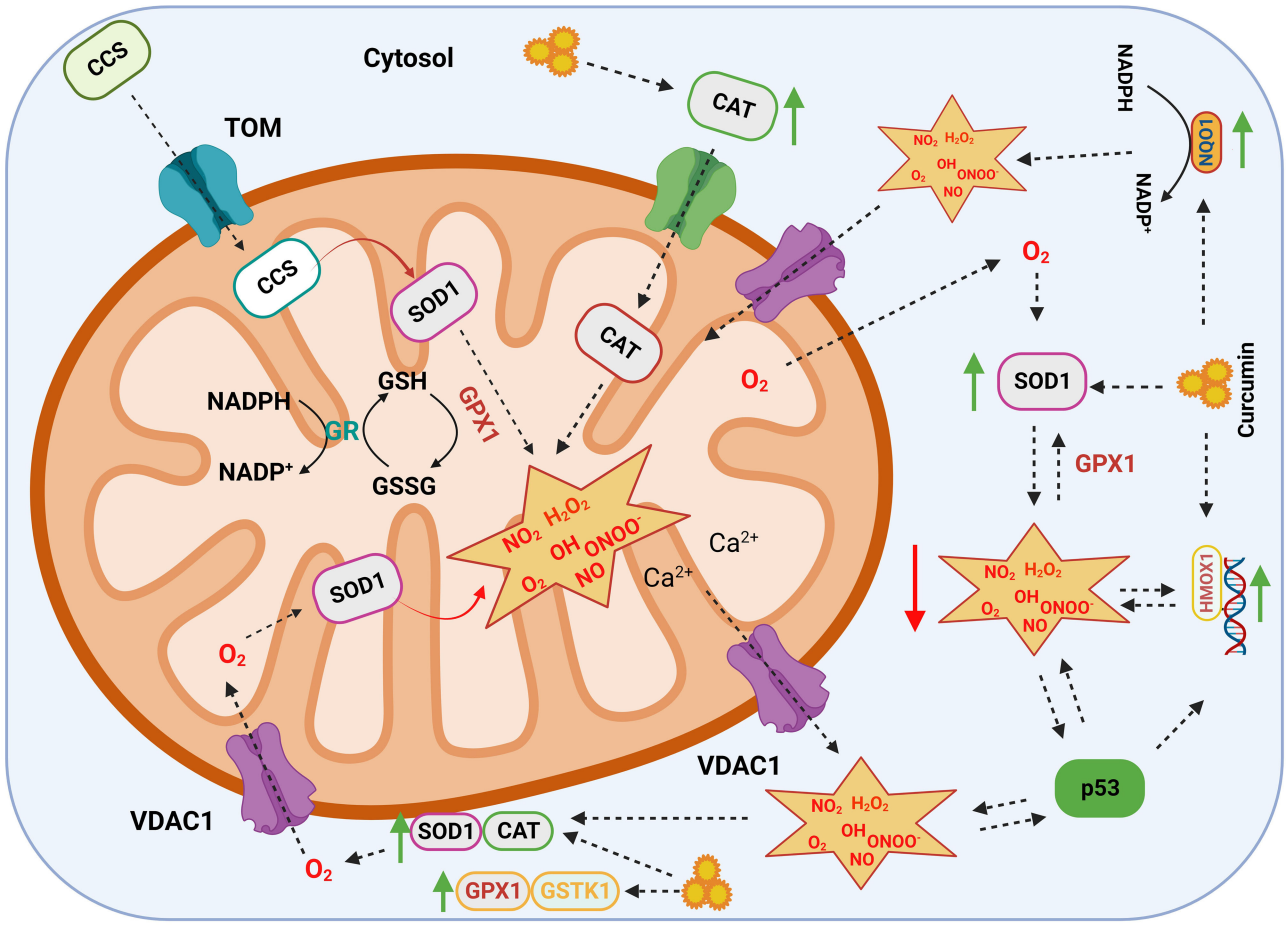
Antioxidant mechanism of curcumin. Schematic diagram summarizing a network of antioxidant enzymes regulating the formation of reactive oxygen species (ROS), which are involved in a number of cellular reactions. Curcumin upregulates antioxidant enzymes such as GPX1, GSTK1, SOD1, CAT, NQO1, and HMOX1. The ROS present in the cytoplasm are transported to the mitochondrion through gatekeeper proteins such as VDAC1 where they are scavenged by antioxidant enzymes. SOD1, superoxide dismutase; CAT, catalase; GPX1, glutathione peroxidase; GSTK1, glutathione-s-transferase kappa; HMOX1, heme oxygenase; NQO1, NAD (P) H quinone dehydrogenase; VDAC1, voltage-dependent anion channel; TP53, cellular tumor antigen p53; CCS, copper chaperone for SOD; GR, glutathione reductase; NO_2_, nitrogen dioxide; H_2_O_2_, hydrogen peroxide; OH, hydroxyl radical; O_2_, superoxide; NO, nitric oxide; ONOO^−^, peroxynitrite. The green arrow represents upregulation and the red arrow represents downregulation.

The anti-inflammatory and antioxidant properties of curcumin are extensively studied by researchers. Curcumin primarily modulates its anti-inflammatory activities by quenching the free radicals *via* NF-κB, TGF-β, and mitogen-activated protein kinase pathways and performs anti-oxidation behavior *via* the Nrf2 pathway (38, 39). In addition to the Nrf2 pathway, curcumin increase the antioxidant capacity by altering antioxidant enzymes (i.e., CAT, SOD1, and GPX1), along with other channel proteins and chaperones (Figure 2).

Curcumin and other curcuminoids are well-known for their functioning on stomach disorders with a remarkable impact on healing various gastric-related difficulties, including ulcer healing (40). A large number of *in vitro, in vivo*, and *in silico* studies have shown that curcumin interactions at the molecular level in multiple signaling pathways (i.e., NF-κB, MAPK/ERK, and STAT) associated with cancers (41). Curcumin is a potential antimicrobial agent. It inhibits the activity of microorganisms such as bacteria, fungi, and viruses by targeting and eventually inactivating growth regulatory genes (42–45). Improving animal nutrition by enhancing the meat quality and increasing weight of the animals, as well as by accelerating their immune system as an initiative to remain disease-free in poultry and livestock animals is also an emerging aspect of curcumin research (46–48). Curcumin's effect on animals is not limited to enhancing growth and improving their physical health but also improves their reproductive health by increasing reproductive performance-related gene expression, which is also a crucial factor in increasing production. For instance, Jiang et al. (49) reported that dietary curcumin supplements resulted an increase in bcl-2 and decrease in caspase-3 gene expression, which in turn alleviate the testicular cell apoptosis in sheep production.

The insecticidal activity of curcumin is another important topic to be explored because curcumin may excellently contribute to the agricultural sector in crop protection and access to non-harmful insecticides from natural products. Curcumin has shown its insecticidal potential by inducing various growth inhibitory activities in insect pests (50, 51). Curcumin and its derivatives may be a possible solution to the protection of vector-borne diseases, particularly diseases spread by mosquitoes such as dengue fever and malaria, is an approach explored by several scientists by evaluating its efficacy *in vivo* (32, 52).

## 3. Curcumin nanoformulations

To overcome curcumin's limitations, such as low bioavailability, poor absorption, and rapid metabolism followed by systemic elimination, scientists have developed various strategies to administer curcumin in the form of a drug and to evaluate its efficacy. Many studies showed that curcumin nanoformulations can be a more effective therapeutic agent than curcumin itself both *in vitro* and *in vivo* (53, 54). Different curcumin nanoformulations are summarized in Figure 1. To begin with, a polymeric nanoparticle encapsulated formulation of curcumin, commonly known as polymeric nanoparticle-encapsulated curcumin (“nanocurcumin”), was reported to have therapeutic efficiency *in vitro* human pancreatic cell lines in a study by Bisht et al. They confirmed that the mechanistic specificity of curcumin remains unchanged in nanocurcumin while reducing the levels of pro-inflammatory cytokines (55). Liposomes are efficient drug delivery agents that enable the transportation of encapsulated compounds to their target sites while minimizing systemic toxicity (56). Nanogel formulations of curcumin are another mode of the highly advantageous delivery system.

Reeves et al. proposed that the curcumin nanogel formulations prepared by directly mixing the curcumin solution in DMSO with aqueous NG127 dispersion and simple post-treatment had excellent tumor cell killing capacity in MDA-231 breast cancer cell lines (57). Curcumin loaded in polymeric micelles is a promising approach to stabilize the compound and utilize it for further pharmacological studies (58). By taking into account the property of magnetic targeting of nanoparticles and Polyethylene glycol (PEG) conjugated drug, Ayubi et al. developed magnetic nanoparticles in which PEGylated curcumin (MNP@PEG-Cur) was used as a surface modifier as a drug delivery system. As a result of various assays and treating mice with different doses of the MNP@PEG-Cur, they concluded that MNP@PEG-Cur is a non-hemolytic, non-toxic material, and all mice were alive without any abnormal behavior (59). The stability and bioactivity of curcumin were reported to be maintained in polysaccharides-based complex particles (60). Kim et al. proved that the nanosphere loaded with curcumin blocks the mitochondrial apoptotic signaling pathway induced by the bacterium *Vibrio vulnificus* in human gastrointestinal epithelial HT-29 cells.

Similarly, nanospheres loaded with curcumin were determined to have cellular uptake by human gut epithelial HCT116 cells and mouse models of gut epithelial migration. It was observed in this study that these nanospheres significantly increased the motility of HCT116 cells and showed much higher migration efficacy than curcumin. Most importantly, nanospheres loaded with curcumin trigger PKC-dependent JNK phosphorylation and were found to regulate the transcriptional activation of NF-κB (61, 62). Curcumin-loaded dendrimers, which are highly branched with potential to manipulate and control separate components, make them suitable candidates for several biomedical applications (63). Similarly, nanoformulations of curcumin with metals and metal oxide particles are a progressing synthesis field of inorganic nanomaterials (64).

Gold nanoparticles are considered excellent drug carriers because of their high loading capacity, stability, and hydrophilicity of drugs (65). Muniyappan et al. synthesized curcumin-capped gold nanoparticles (CUR-AuNPs) through green synthesis. The CUR-AuNPs exhibited antioxidant, anti-inflammatory, and antibacterial properties effectively, which were confirmed with the help of treating the CUR-AuNPs on hydrogen peroxide, human red blood cells, and four different bacteria species, respectively (66). Recently, a study by Piwowarczyk et al. used a modified thin-film hydration method to embed curcumin and a mixture of other polyphenols in the liposomal nanoformulation to study its stability and anti-cancer potential (67). Despite the extensive researches on curcumin nanoformulation in biomedical research, no research has been performed about the effect of curcumin nanoformulation on livestock and animal production, with a few notable exceptions wherein curcumin nanospheres were supplemented in the diet of weaned piglets (23).

## 4. Curcumin's role in increasing livestock and poultry production performances

In recent years, livestock and poultry industries have mainly targeted the introduction of balanced diets to enhance the growth of animals and increase the resistance to diseases, over-all health conditions of animals, and environment-friendly production. In this view, plant-based natural feed additives improve livestock and poultry production performance, does not cause any side effects for animals, and ensure the safety of products for human consumption. Researchers suggest that plant-based natural products like curcumin are promising replacement additives to antibiotics (68). In this section, we discuss how curcumin contributes to improving livestock and poultry animal production.

### 4.1. The impact of curcumin on livestock performance

Researchers suggest that farmers prioritize the minimization of health issues in livestock. At the same time, farmers want the animals to be naturally healthy with their own individual characteristics (69). While taking into account this demand of farmers, as a matter of fact, natural compounds as a substitute for improving livestock health can be a practical step to provide a better safety profile while taking care of animal health (70).

#### 4.1.1. Antimicrobial, antioxidant and anti-inflammatory effects

Curcumin reduces *E. coli-*induced generation of inflammatory mediators such as IL-1 while increasing antibody secretion at modest doses (300 mg/kg) (71). In addition, Gan et al. (72) showed that curcumin decreases copy number of pathogenic bacteria *E. coli* in the gut, downregulates TLR4 signaling pathways, adjust of interleukin levels, and elevates immunoglobulin levels in weaned piglets. These properties are all advantageous for piglet's growth and development during weaning when curcumin and resveratrol are ingested orally in the feed (68). *Besnoitia besnoiti* is a protozoan parasite causing a reduction in cattle's, reducing fertility and productivity and consequently, leading to economic losses. The antiparasitic efficacy of curcumin checked in *in vitro* bovine epithelial cell lines demonstrated that curcumin pretreatments of tachyzoites resulted in a dose-dependent reduction of host cell invasion and is safe and beneficial to be included in the cattle feed (73).

In Hu sheep (rare localized Chinese sheep breed), a dietary curcumin supplement was investigated, and it was found to promote lipid metabolism, antioxidant status, reproductive performance and improve immune ability by increasing the concentrations of IgA, IgM, and IgG in plasma (49). Meanwhile, nursing lambs were examined for body weight gain through stimulating creatine kinase activity and preventing fat decrease of ATP content (74). A whole transcriptomic *in vitro* study by Pauletto et al. validated that curcumin reduces AFB1-induced hepatic toxicity by triggering molecular pathways related to anti-inflammatory and antioxidant responses in cattle (75).

The well-developed and disease-free gut systems of animals play a vital role in maintaining good health. IUGR is a major crisis to be addressed to enhance good health in the livestock industry. Based on the antioxidative ability of curcumin on livestock animals, curcumin alleviated IUGR jejunum damage in pigs through Nrf2/Keap1 pathway while given as a feed additive in such a way that oxidative stress is reduced along with intestinal development (76, 77). Likewise, Li et al. reported that curcumin improved oxidative stress conditions in jejunum cell organelles and membranes in piglets (78). A similar study by Zhang et al. suggests that curcumin improves meat quality in pigs. This finding implies that curcumin may function as a natural antioxidant in IUGR offspring diet interventions to improve the meat quality and redox status of leg muscles (79). Shi et al. showed that piglet feed had more beneficial effects when curcumin was administered with piperine (80). Curcumin nanospheres supplemented in weaned piglet diets were reported to enhance growth, feed utilization, and immunity and reduce fecal pathogenic bacteria and ammonia gas emissions (23).

#### 4.1.2. Intestinal health

In piglets infected with *E. coli*-induced intestinal injury, curcumin repairs, and improves the morphology of ileum epithelial mucosa (71). Curcumin and resveratrol enhanced the intestinal antioxidative capacity in weaning piglets by increasing the mRNA expression levels of tight junction proteins (68). Pauletto et al. suggested that curcumin help to mitigate jejunum injury by regulating the antioxidant capacity through the Nrf2/Keap1 pathway. This pathway enhanced the jejunum function by improving the immune function and jejunal tight junction in IUGR pigs (75).

#### 4.1.3. Animal reproductive health

The reproductive health of livestock animals includes many aspects, such as a healthy gestation period, fetal health, milk production, etc. Dairy cows supplemented with certain phytonutrients (condensed tannins, encapsulated cinnamaldehyde, capsaicin, and piperine), including curcumin, have improved milk production and nutritional status in ¾ Holstein × ¼ Gir cows (81). Dietary curcumin improved the testosterone levels and testicular volume in Baladi bucks in the non-breeding season. Baladi bucks are goat bucks, seasonal breeders with very low fertilizing potentials, and other reproductive behaviors. Therefore, curcumin can be solution to overcome breeding inabilities in such a rarely reproducing dairy animal is a further opening to a more advanced level of research (82). The reported effects of curcumin in livestock animals are summarized in Table 1.

**Table 1:** The described effects of curcumin supplementation in livestock animals.

**S. No**.	**Study**	**Effective dosage**	**Animal**	**Main findings**	**References**
1	Effects of curcumin on growth performance, jejunal mucosal membrane integrity, morphology, and immune status in weaned piglets challenged with enterotoxigenic *Escherichia coli*	300–400 mg/kg	*Sus scrofa domesticus* (Pig)	Curcumin acts as an alternative for the antibiotic quinocetone in diets fed to weaned piglets by improving their health and growth status	(71)
2	Curcumin and resveratrol regulate intestinal bacteria and alleviate intestinal inflammation in weaned piglets	300 mg/kg	*Sus scrofa domesticus* (Pig)	Curcumin enhances intestinal immune function by regulating the piglet gut microbiota and decreasing intestinal inflammation *via* down-regulating TLR4 signaling pathway	(72)
3	Antiparasitic efficacy of curcumin against *Besnoitia besnoiti* tachyzoites *in vitro*	5.93 μM	*Bos taurus* (Cattle)	Curcumin reduced *Besnoitia besnoiti* tachyzoites viability with up to 56% mortality. Hence curcumin has anticoccidal activity *in vitro*	(73)
4	Curcumin supplement in summer diet on blood metabolites, antioxidant status, immune response, and testicular gene expression in Hu sheep	450 and 900 mg/sheep/day	*Ovis aries* (Hu sheep)	Dietary curcumin supplementation (450 and 900 mg/per sheep daily) can promote lipid metabolism, antioxidant capacity, and immune response as well as testicular development in Hu sheep	(49)
5	Diet supplemented with curcumin for nursing lambs improves animal growth, energetic metabolism, and performance of the antioxidant and immune systems	100–200 mg/kg	*Ovis aries* (Lamb)	Curcumin enhanced enzyme activity which then lead to anti-inflammatory action and weight gain in lambs	(74)
6	Productive and physiological responses of lactating dairy cows supplemented with phytogenic feed ingredients	15 g Actifor pro mix	*Bos Taurus taurus* × *Bos primigenius indicus* (Holstein × Gir cows)	Curcumin in the presence of other phytocompounds improved milk production and enhanced nutritional status	(81)
7	Curcumin mitigates AFB1-induced hepatic toxicity by triggering cattle antioxidant and anti-inflammatory pathways: A whole transcriptomic *in vitro* study	450 mg/kg in feed 10 μM in BFH12 cell lines	*Bos taurus* (Cattle)	Curcumin reduced AFB1 induced toxicity and decreased cells mortality by 30% in bovine fetal hepatocyte-derived cell line (BFH12)	(75)
8	Curcumin alleviates IUGR jejunum damage by increasing antioxidant capacity through Nrf2/Keap1 pathway in growing pigs	200 mg/kg	*Sus scrofa domesticus* (Pig)	Dietary curcumin reduced intrauterine growth retardation jejunum damage in pigs	(76)
9	Dietary supplemented curcumin improves meat quality and antioxidant status of intrauterine growth retardation growing pigs *via* Nrf2 signal pathway	200 mg/kg	*Sus scrofa domesticus* (Pig)	Curcumin served as a natural antioxidant and improved the meat quality, redox status, and growth performance	(79)
10	Effect of the single and combined use of curcumin and piperine on growth performance, intestinal barrier function, and antioxidant capacity of weaned Wuzhishan piglets	200 and 300 mg/kg	*Sus scrofa domesticus* (Wuzhishan Pig)	Curcumin improved intestinal permeability and reduced oxidative stress	(80)
11	Evaluation of dietary curcumin nanospheres in a weaned piglet model	0.5 and 1.0 ml solutions of curcumin nanospheres	*sus scrofa domesticus* [Duroc × (Yorkshire × Landrace)]	Curcumin nanospheres reduce fecal pathogenic bacteria, ammonia gas emissions in weaned piglets along with enhancing their growth, immunity and feed utilization	(23)
12	Supplemental dietary curcumin improves testicular themodynamics, testosterone levels, and semen quality in Baladi bucks in the non-breeding season	200 mg/kg	*Capra aegagrus hircus* (Goat)	Curcumin improved reproductive factors such as testosterone levels and testicular volume	(82)
13	Effects of curcumin on mitochondrial function, endoplasmic reticulum stress, and mitochondria-associated endoplasmic reticulum membranes in the jejunum of oxidative stress piglets	200 mg/kg	*Sus scrofa domesticus* (Pig)	Curcumin prevented mitochondria-associated endoplasmic reticulum (ER) membranes (MAMs) disorder in oxidative stress piglets	(78)

### 4.2. The impact of curcumin on poultry performance

Avian meat and other consumables are the major sources of nutrients for humans. Therefore, the use of antibiotics for poultry is increased to improve poultry production. Antibiotics may cause direct and indirect negative effects on animal and human health (83). Moreover, the massive use of antibiotics to promote growth may cause the development of antibiotic resistance in animals and affect the health of animals and consumers (84). Through dietary formulation strategies, numerous natural compounds provide the ability to support the health of poultry while improving the nutritional quality of meat and eggs as well (1). So far, different natural compounds have been evaluated as feed additives in the poultry industry (85–87). Curcumin is one of those feed additives, considered to be an excellent non-toxic feed additive that improves the immunity, growth performance, and behavioral patterns of poultry animals (88).

#### 4.2.1. Heat and oxidative stress

Dietary curcumin was reported to prevent growth impairment induced by heat stress in broilers (89). It was observed that curcumin supplementation increased levels of various detoxifying enzymes (Glutathione peroxidase, glutathione S-transferase, and manganese superoxide dismutase) and decrease (*P* < 0.05) of heat shock protein 70 mRNA levels in the breast muscle. Similarly, it was also shown that curcumin activated the glutathione peroxidase and Nrf2-mediated phase II detoxifying enzyme systems simultaneously in poultry to scavenge reactive oxygen species, which caused oxidative stress (90). Likewise, curcumin increased the mitochondrial manganese superoxide dismutase gene expression and mitigated the hepatic mitochondrial dysfunction in heat-stressed broilers. Nawab et al. observed increased antioxidant enzyme expression levels, similar to Zhang et al. (90), in heat-stressed laying hens when administered with curcumin-supplemented feed (91). Curcumin exhibited positive responses on antioxidant capacity, lesion score, and oocyst shedding in a dietary treatment study conducted by Yadav et al. on broiler chickens (48). Due to high stocking densities under stressful conditions in broilers, the increase in the mRNA expression levels of insulin-like growth factor-1 (IGF-1), growth hormone receptor (GHR), myostatin (MSTN), and leptin in liver tissues enhancement in curcumin supplementation were noticed by Hafez and coworkers showed curcumin's oxidative stress-relieving ability and potential of increasing poultry growth performance and immune status (88).

#### 4.2.2. Protective effect on poultry gut microbiome

The interplay of curcumin and the gut microbiota of poultry is an increasing area of research since curcumin has protective and antagonistic effects against both beneficial and harmful microbes, respectively (47). A solid dispersion form of curcumin and boric acid has a synergistic bactericidal impact against a bacterial poultry disease potentially causing foodborne illness in humans when consumed (92). Curcumin also has a protective effect against liver oxidative injury in ducks by lipid metabolism disruption by modulating intestinal microbiota (47). According to Yadav et al. (48) certain species of gut microbiota located in different sites play a major role in maintaining intestinal integrity in poultry. They found that *Eimeria* spp (parasites that cause coccidiosis in poultry) infected broilers had a leaky gut due to infection caused by the parasite infection. However, curcumin-fed birds had lower gut permeability at 100 mg/kg doses than other experimental group birds (48). The antimicrobial activity is another primary advantage in protecting the poultry from harmful disease-causing microbes *via* ingesting curcumin in the animal feed (93).

#### 4.2.3. Egg and meat quality

Improving meat and egg quality contribute to economically promoting the poultry sector. Zhang et al. (94) showed that curcumin, as a potential antioxidant, improved meat quality and oxidant stability of muscle in broilers. In a similar study, feed-added curcumin was investigated to decrease the total cholesterol and fat content in the breast meat of broiler chicken (95). It is well-known that curcumin has a scavenging effect over oxidative stress because of its antioxidant capability, which also becomes advantageous when supplemented in poultry feed in several ways (96–98). Liu and coworkers shows that, on heat-stressed hens, curcumin had a favorable impact on laying performance and egg quality. Curcumin supplementation improves laying performance and egg quality by significantly increasing egg production, egg shell thickness, eggshell strength, and albumen height while decreasing the feed-to-egg ratio (99). Additionally, spraying curcumin in different concentrations on hatching eggs of dokki-4 chickens (a breed of chicken native to Egypt) was examined after exposure to thermal stress. Best hatchability rates and other productive traits were improved in eggs sprayed with curcumin compared to the non-sprayed eggs (100). The addition of curcumin to broiler diets significantly improved the levels of monounsaturated fatty acids (MUFAs) and polyunsaturated fatty acids (PUFAs) in breast and thigh muscles (101). Similarly, a recent study showed that egg production in broilers benefited from improved oxidative stability and egg shell breaking force when a phytonutrient solution of *Curcuma* was supplemented in laying hens (102). Curcumin supplementation in duck feed improved the meat quality by limiting the extent of lipid oxidation during the post-mortem period (103).

#### 4.2.4. Detoxifying effects

Aflatoxins (AFB1) are toxic metabolites that reduce productivity and reproductive performance, eventually leading to organ malfunctions (104). Curcumin's detoxifying effect against aflatoxins in poultry is a well-studied topic that has cited various protective mechanisms of the same (105, 106). In an *in vivo* exposure model study carried out upon arbor acres broiler, curcumin successfully inhibited a cytochrome P450 enzyme that mediated the bioactivation of AFB1 (107). AFB-1-induced toxicity was reported to be reduced by curcumin in combination with another cellulosic polymer in broiler chicken (108). AFB1-induced spleen damage was regulated by curcumin by activating the Nrf2 signaling pathway, upregulating the expression of antioxidant enzymes, and inhibiting the NF-κB pathway in ducklings (109). Curcumin also initiate a reduction in the toxicity of AFB1 by activating the Nrf2-ARE signaling pathway and inhibiting the NF-κB signaling pathway in a study conducted on ducks that investigated acute ileum damage (110). Meanwhile, curcumin administration attenuated the renal oxidative stress parameters induced by AFB1 in broiler chickens. The stress parameters evaluated in this study included serum antioxidant capacity and enzymatic activity of kidney superoxide dismutase, catalase, and glutathione peroxidase, which were found to be downregulated (97).

On the other hand, curcumin can also protect against AFB1-induced necroptosis and inflammation by regulating TLR4/RIPK pathway in arbor acres broilers (111). Recently, Ruan et al. reported that curcumin modulated LPS-induced homeostatic imbalance by modulating gut microbiota through the BA-FXR pathway in chickens (112). Tang et al. observed curcumin's effect on arsenic-induced toxicity by growth inhibition, reduced hyaline degradation, and distortion in duck spleen, along with suppression of various pro-inflammatory cytokines and autophagy-related genes (113). In addition to aflatoxins, curcumin may protect ducks from ochratoxin, a mycotoxin that is difficult to remove from feed and induces impairment of intestinal barrier function and mitochondrial integrity (114).

The protective effect of curcumin against ochratoxin-A-induced liver injuries in the duck was evaluated by Zhai et al. They found that curcumin supplementation relieved the decreased abundance of butyric acid-producing bacteria which were induced by ochratoxin-A (47). The above-mentioned studies suggest curcumin potentially increase meat quality, especially in broiler chickens, the most commonly consumed avian product. Curcumin effect in poultry animals is described in Table 2.

**Table 2:** The described effects of curcumin supplementation in poultry animals.

**S. No**.	**Study**	**Effective dosage**	**Animal**	**Main findings**	**References**
1	Dietary curcumin supplementation protects against heat-stress-impaired growth performance of broilers possibly through a mitochondrial pathway	100 and 200 mg/kg	*Gallus gallus domesticus* (Broiler chicken)	Curcumin prevented growth impairment due to heat stress by improving the antioxidant defense system and enhancing the mitochondrial biogenesis	(89)
2	Effect of various levels of dietary curcumin on meat quality and antioxidant profile of breast muscle in broilers	50 and 100 mg/kg	*Gallus gallus domesticus* (Broiler chicken)	Curcumin improved meat quality and muscle oxidant stability with its antioxidative properties	(94)
3	Curcumin successfully inhibited the computationally identified CYP2A6 enzyme-mediated bioactivation of Aflatoxin B1 in arbor acres broiler	450 mg/kg	*Gallus gallus domesticus* (Arbor Acres broiler)	Curcumin inhibited an enzyme associated with AFB1 bioactivation in arbor acres broilers	(107)
4	Dual role of dietary curcumin through attenuating AFB1-induced oxidative stress and liver injury *via* modulating liver Phase-I and Phase-II enzymes involved in AFB1 bioactivation and detoxification	450 mg/kg	*Gallus gallus domesticus* (Arbor Acres broiler)	Preventive actions of curcumin against AFB1-induced liver injury in broilers	(106)
5	Curcumin attenuates heat-stress-induced oxidant damage by simultaneous activation of GSH-related antioxidant enzymes and Nrf2-mediated phase II detoxifying enzyme systems in broiler chickens	100 and 200 mg/kg	*Gallus gallus domesticus* (Broiler chicken)	Curcumin positively modulated antioxidant enzyme GSH and antioxidant activity related pathway	(90)
6	Evaluation of a solid dispersion of curcumin with polyvinylpyrrolidone and boric acid against *Salmonella Enteritidis* infection and intestinal permeability in broiler chickens: a pilot study	0.05 and 0.1% CUR/PVP	*Gallus gallus domesticus* (Broiler chicken)	Curcumin possessed antimicrobial effects against *Salmonella Enteritidis*, a bacterial disease of poultry	(92)
7	Effect of dietary curcumin on the antioxidant status of laying hens under high-temperature conditions	200 mg/kg	*Gallus gallus domesticus* (Hen)	Curcumin increased expression levels of antioxidant enzymes in laying hens under heat stress	(91)
8	Evaluation of cellulosic polymers and curcumin to reduce Aflatoxin B1 toxic effects on performance, biochemical, and immunological parameters of broiler chickens	0.2 %	*Gallus gallus domesticus* (Broiler chicken)	Detoxifying effects of curcumin from AFB1 toxins in broilers	(108)
9	Effects of curcumin on performance, antioxidation, intestinal barrier and mitochondrial function in ducks fed corn contaminated with ochratoxin A	400 mg/kg	*Anas platyrhynchos domesticus* (Pekin ducks)	Curcumin reduced enterotoxicity caused by ochratoxin A, a naturally occurring food borne mycotoxin and various other intestinal health parameters	(114)
10	The effects of different doses of curcumin compound on growth performance, antioxidant status, and gut health of broiler chickens challenged with *Eimeria* species	200 mg/kg	*Gallus gallus domesticus* (Broiler chicken)	Curcumin had effective improvement against *Eimeria* infection	(48)
11	Protective effect of curcumin on ochratoxin A–induced liver oxidative injury in duck is mediated by modulating lipid metabolism and the intestinal microbiota	400 mg/kg	*Anas platyrhynchos domesticus* (Pekin ducks)	Curcumin modulated the cecum microbiota in ducks and lessened lipid metabolism and OTA-induced injury	(47)
12	Feed added curcumin with increased solubility on plasma lipoprotein, meat quality, and fat content in broiler chicks	0.2%	*Gallus gallus domesticus* (Broiler chicken)	Curcumin reduced total cholesterol and fat content of broiler chicken breast meat	(95)
13	Effect of curcumin on laying performance, egg quality, endocrine hormones, and immune activity in heat-stressed hens	150 mg/kg	*Gallus gallus domesticus* (Hen)	Curcumin supplementation in hen diet improved egg quality and laying performance	(99)
14	Evaluation of curcumin and copper acetate against *Salmonella Typhimurium* infection, intestinal permeability, and cecal microbiota composition in broiler chickens	1:9 ratio of curcumin in polyvinylpyrrolidone	*Gallus gallus domesticus* (Broiler chicken)	Curcumin displayed its antimicrobial effect and protect broiler chickens from hazardous microbes	(93)
15	dietary curcumin alleviated acute ileum damage of ducks (*Anas platyrhynchos*) induced by AFB1 through regulating Nrf2-ARE and NF-κB signaling pathways	500 mg/kg	*Anas platyrhynchos* (Duck/Mallard)	Antioxidation and anti-inflammatory activity of curcumin protected the ileum of ducks *via* activating Nrf2-ARE signaling pathway and inhibiting NF-κB signaling pathway	(110)
16	Dietary curcumin improves energy metabolism, brain monoamines, carcass traits, muscle oxidative stability and fatty acid profile in heat-stressed broiler chickens	100 mg/kg	*Gallus gallus domesticus* (Broiler chicken)	Curcumin increased MUFA and PUFA content in breast and thigh muscles of broilers	(101)
17	Impact of treating hatching eggs with curcumin after exposure to thermal stress on embryonic development, hatchability, physiological body reactions, and hormonal profiles of Dokki-4 chickens	250 mg/litter	*Gallus gallus domesticus* (Dokki-4 chicken)	Curcumin treatments triggered better hatchability traits	(100)
18	Curcumin supplementation protects broiler chickens against the renal oxidative stress induced by the dietary exposure to low levels of Aflatoxin B1	400 mg/kg	*Gallus gallus domesticus* (Broiler chicken)	Curcumin diminished oxidative stress caused by AFB1 in chicken kidney	(97)
19	The impact of curcumin on growth performance, growth-related gene expression, oxidative stress, and immunological biomarkers in broiler chickens at different stocking densities	200 mg/kg	*Gallus gallus domesticus* (Broiler chicken)	Curcumin increased mRNA expression levels of IGF-1, GHR, MSTN	(88)
20	Curcumin alleviates LPS-induced intestinal homeostatic imbalance through reshaping gut microbiota structure and regulating group 3 innate lymphoid cells in chickens	300 mg/kg	*Gallus gallus domesticus* (Broiler chicken)	Curcumin modulated homeostatis imbalance in gut microbiota through BA-FXR pathway	(112)
21	The association of curcuma and Scutellaria plant extracts improves laying hen thermal tolerance and egg oxidative stability and quality under heat stress conditions	1:1 ratio of CUR and SCUT	*Gallus gallus domesticus* (Hen)	Curcuma supplementation improved oxidative stability and egg shell breaking force	(102)
22	Curcumin activates the Nrf2 pathway to alleviate AFB1-induced immunosuppression in the spleen of ducklings	400 mg/kg	*Anas platyrhynchos* (Ducklings)	Curcumin activated Nrf2 signaling pathway, upregulated the expression of antioxidant enzymes and inhibited NF-κB pathway in ducklings with AFB-1 induced spleen damage	(109)
23	Protective role of curcumin on aflatoxin B1-induced TLR4/RIPK pathway mediated-necroptosis and inflammation in chicken liver	300 mg/kg	*Gallus gallus domesticus* (Broiler chicken)	Curcumin reduced inflammatory cytokines levels, oxidative stress biomarkers, inflammation genes triggered by AFB1-induced necroptosis through TLR4/RIPK pathway	(111)
24	Curcumin antagonizes infammation and autophagy induced by arsenic trioxide through immune protection in duck spleen	400 mg/kg	*Anas platyrhynchos* (Sanshui white ducks)	Arsenic-induced toxicity was controlled by reduced hyaline degradation and distortion in duck spleen, suppression of various pro-inflammatory cytokines and autophagy-related genes	(113)

## 5. Curcumin's protective effect against insect pests

Class Insecta is the most species-rich animal category on the planet. Some insects may cause deleterious effects in to humans, poultry and livestock animals, and crops. The increasing rate of mutation in insects due to insecticide resistance, frequent use of insecticides and other various climatic and environmental factors has a bearing on many aspects of human health (115–117). Advancing the subject of how insects cause turmoil to animals, insects are vectors of many contagious parasitic diseases, especially insects that feed on blood from animals can have adverse effects on the wellbeing, behavior, and productivity of livestock animals (118). In addition, economic losses caused by these behavioral changes are high (119). Chemical insecticides are extensively used to control pest insects. The detrimental aspect of chemical insecticides include harmful effects in disturb human health and the environment (120). This negative effect drives a resurgence of interest in insecticides based on natural compounds because of their minimal costs and ecological side effects (121). Nevertheless, as discussed in earlier sections, natural polyphenols provide a promising source for insecticidal applications (28). Kindly note that curcumin is one such polyphenol (30–32). Researchers have explained the anti-insect effects and safety of curcumin to different insects. In the following sections, damage caused by insect pests in the agriculture sector and animal health and wellbeing is outlined and discussed, along with how curcumin plays a role in eradicating those destructive complications.

### 5.1. Vector mosquitoes

The *A. aegypti* and *A. albopictus* are the most common species responsible for the transmission of the deadly vector-borne virus disease, dengue (122). The research outcome from de Souza et al. (123) indicates that curcumin in sugar formulations is highly efficient, proving it to be a promising and safe alternative to control *A.aegypti* mosquitoes. This study evaluated curcumin's photolarvicidal and ovicidal activity in sucrose and D-mannitol. According to Raman microspectroscopy results, D-mannitol showed high permeability to the larva's peritrophic membrane, causing irreversible damage to the simple columnar epithelium of the digestive tube (123). Relatedly, curcumin formulated has photodynamic activity against *A.aegypti* larvae and yielded a water-soluble, non-toxic byproducts of curcumin as a result of photodegradation (124). In a larvicidal bioassay study with the integration of *in silico* molecular structural validations in *C.pipiens* and *A. albopictus*, curcumin derivatives appeared to have larvicidal effects. Among the tested compounds, four exhibited a mortality rate ≥10% in both *C. pipiens* and *A. albopictus* larvae, followed by evaluation of larvicidal activity using various concentrations for 24 h incubation (32). An *in-silico* study followed by *in vivo* and *in vitro* validations by the research group of Rao et al. revealed curcumin's molecular interplay in controlling *C. pipiens*. Rao et al. revealed in *C. pipiens* that curcumin increase the mortality in the mosquitoes vector at an early stage in its life cycle by Acetylcholine esterase 1 (AchE1) inhibition. *In vitro* and *in vivo* inhibition assays shows that AChE inhibitor pyridostigmine bromide and malathion were used as controls for examining the AChE inhibition by curcumin from the larvicidal extract. The authors also claim that curcumin induced larvicidal activity by employing competitive inhibition of AChE, hence curcumin may be a potential replacement for carbamates and organophosphates, popular AChE inhibitor (125).

### 5.2. Agricultural insect pests

Agricultural insect pests controlled by botanicals from *C. longa*, including essential oils, chemical constituents, and other extracts, are known to be studied for their role as “crop protectors” (126). Hemanta Chowdhury et al. (127) isolated and characterized turmeric components and derivatives, which were shown to have a modest insect growth inhibitory effect on *Schistocerca gregaria* (Forsk) and *Dysdercus koenigii* (Walk). These conclusions were made based on the inhibitory activities of the compounds in both insect species; however, they found that turmeric oil had a nymphal mortality rate of 60%, along with 10% abnormal growth compared to the test compounds. Therefore, turmeric oil was insecticidal rather than growth-inhibiting. They concluded that curcumin-I, its dibutyl derivative (7), and the benzene extract of turmeric rhizome powder, which contains both turmerones and curcuminoids, were the most active (127).

*Tetranychus cinnabarinus* or carmine spider mites are also catastrophic agricultural pests. Transcriptomics and functional enrichment analysis by Liu et al. (50) revealed 23 differentially expressed genes that were functionally identical or similar to the targets of insecticide/acaricides or genes that were associated with mite detoxification and metabolism. In particular, calmodulin, phospholipase A_2_, and phospholipase C were activated upon curcumin treatment suggesting that the calcium channel related genes might play important roles in mite's response to curcumin (50).

**Figure 3:**
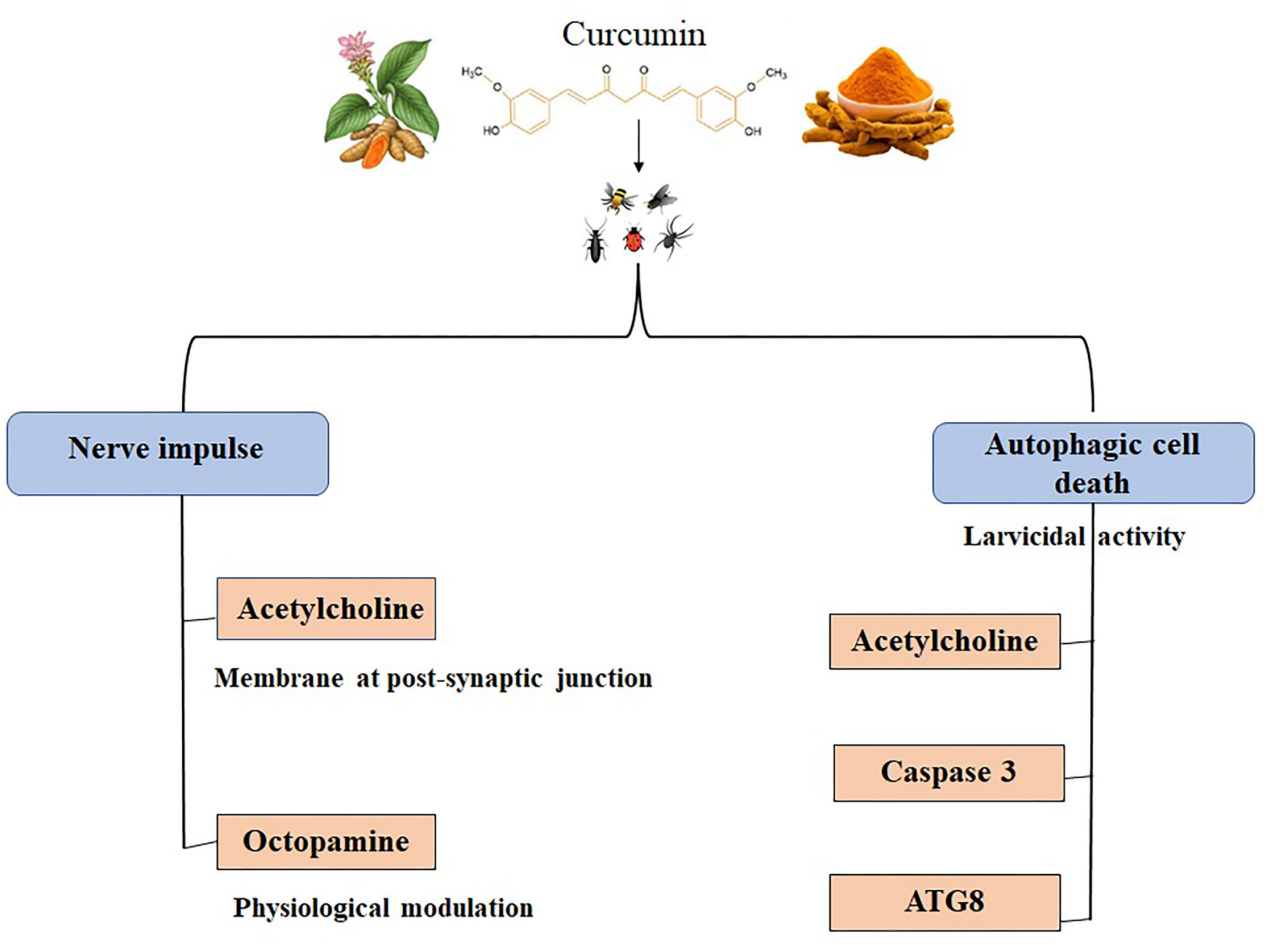
Insecticidal effects of curcumin. Acetylcholine and octopamine are the neurotransmitters in insects affected by curcumin, leading to blockage of nerve impulse. Acetylcholine, caspase 3, and ATG8 are targeted while curcumin induces autophagic cell death in insect larvae (123, 124, 128).

Lepidopterans are one of the most widely studied agricultural pest categories partly because of their ability to absorb and digest nutrients rapidly. Along with the fact that environmental microorganisms easily enter the gut system of lepidopterans, enhancing their crop destruction capabilities (128). Veeran et al. (31) reported the cytotoxic effect of curcumin in insect cells for the first time, wherein curcumin induced autophagic cell death in a time and dose-dependent manner in a lepidopteran *S. frugiperda in vitro* cell lines. They observed that the autophagy induction effect of curcumin using various morphological assays and cell proliferation assays in Sf9 cells. In addition, autophagy-related proteins, ATG8-I, and ATG8-II expression levels were elevated after curcumin treatment. Curcumin and avermectin synergistic effects against *S.litura* tested at *in vitro* and *in vivo* condition. It revealed that programmed cell death was may be the possible synergistic mechanism of curcumin combined with avermectin (129). Curcumin elevated the expression of xanthotoxin-induced detoxification genes by regulating ROS/CncC and signaling pathway genes in *S. Litura* (130). Interestingly, CnCC is the insect ortholog of the transcription factor, Nrf2, which has been mentioned in previous sections to have been studied to be regulated by curcumin (131). Curcumin's effect on insects is depicted in Figure 3 (123, 124, 128) and Table 3.

**Table 3:** Curcumin's protective effect against different insect pests.

**S. No**.	**Study**	**Effective dosage**	**Insect**	**Main findings**	**References**
1	Isolation, characterization and insect growth inhibitory activity of major turmeric constituents and their derivatives against *Schistocerca gregari*a (Forsk) and *Dysdercus koenigii* (Walk)	20 μg (*Schistocerca Gregaria*), 50 μg (*Dysdercus koenigii*)	*Schistocerca Gregaria* (Forsk), *Dysdercus koenigii* (Walk)	Insect growth inhibitory activity of curcumin-I against *Schistocerca gregaria* and *Dysdercus koenigii* nymphs	(127)
2	Curcumin induces autophagic cell death in *Spodoptera frugiperda* cells	5–15 μg/mL	*Spodoptera frugiperda* (Fall armyworm)	Curcumin induces autophagic cell death in *Spodoptera frugiperda* insect cell line	(31)
3	Curcumin in formulations against *Aedes aegypti*: mode of action, photolarvicidal and ovicidal activity	0.01 and 0.02 mg/l	*Aedes aegypti* (Yellow fever mosquitoes)	Photolarvicidal and ovicidal activity of curcumin by invading and injuring the intestinal epithelium of the larvae	(123)
4	Revealing the molecular interplay of curcumin as *Culex pipiens* Acetylcholine esterase 1 (AChE1) inhibitor	100 ppm	*Culex pipiens* (Northern house mosquitoes),	Curcumin inhibits AChE1 at an early life stage in *Culex pipiens* and induces mortality	(125)
5	Curcumin derivatives as potential mosquitoes larvicidal agents against two mosquitoes vectors, *Culex pipiens* and *Aedes albopictus*	6.0 ppm (*Culex pipiens*) and 9.2 (*Aedes albopictus*)	*Culex pipiens* (Northern house mosquitoes), *Aedes albopictus* (Asian tiger mosquitoes)	Curcumin, demethoxycurcumin, curcumin-BF2 complex and a monocarbonyl tetramethoxy curcumin derivative exhibited high larvicidal activity against *Culex pipiens* and *Aedes albopictus*	(32)
6	Synergistic effects of botanical curcumin-induced programmed cell death on the management of *Spodoptera litura* Fabricius with avermectin	10/1 μg/mL avermectin/curcumin mixed regent	*Spodoptera litura* Fabricius (Cutworm)	Curcumin has synergistic effects toward the pesticide avermectin by inducing programmed cell death	(129)
7	Environmentally safe photodynamic control of *aedes aegypti* using sunlight-activated synthetic curcumin: photodegradation, aquatic ecotoxicity, and field trial	4.2 mg/l	*Aedes aegypti* (Yellow fever mosquitoes)	Curcumin can act as an environment-friendly photosensitizer to control *A. aegypti* larval population by promoting oxidative storms *via* the photodynamic effect	(124)
8	Activation of the ROS/CncC and 20-hydroxyecdysone signaling pathways is associated with xanthotoxin-induced tolerance to λ-cyhalothrin in *Spodoptera litura*	0.2% in artificial diet	*Spodoptera litura* (Cutworm)	Curcumin is a CncC agonist and activated 20E signaling pathway	(130)

Agricultural plant parasite nematodes are microscopic roundworms that live in the soil and feed on plant roots. Many commercially important crops are susceptible to considerable size and quality losses due to plant-parasitic nematodes (138). Infectious disease in agricultural crops due to nematodes is also increasing (139). Curcumin's nematicidal effects have not been well-explored, but there is evidence that turmeric extracts are efficacious. In a study by Rashid et al. utilizing various fractions and concentrations of turmeric, they caused larvicidal mortality in the root-knot nematode *Meloidogyne incognita* (140). Turmeric extract using methanol as a solvent at a concentration of 20% was found to have the most efficacy in suppressing *Meloidogyne* spp (141). 0.5, 1, and 2% concentrations of turmeric extract were used in the pot experiment containing tomato plants with nematode eggs. Plants treated with turmeric extracts had the lowest number eggs in their roots. Mortality rate of second-stage juvenile eggs was higher in 2% concentration of the extract (132).

## 6. Model insects and organisms

Interestingly, model insects and organisms including *Drosophila melanogaster, Apis mellifera*, and *Caenorhabditis elegans* are used to test the curcumin's various beneficial effects. Curcumin was observed to extend lifespan of honeybee. Curcumin decreased global DNA methylation levels and increased the natural age-related level in older bees, along with being an effective natural bio-stimulator, improving apian health and vitality (132). Similarly, lifespan shortening in honeybees (*Apis mellifera*) due to ethanol intake was restored by curcumin when co-administered with ethanol (136). Bees fed with 1:1 *v/v* sugar water with 100 ppm curcumin lowered viral loads by positively impacting the gut microbiome. Curcumin treatment lead to lower levels of *Gilliamella*, a honeybee bacterial gut symbiont (137). 0.2 mg/g curcumin diet fed Drosophila alleviated the increased oxidative stress caused by heat stress, by increasing the expression of SOD1, CAT, and PHGPx and decreasing the expression of Hsp70 and Hsp83 (133). Ten micrometer curcumin, in combination with another phytochemical thymoquinone had significant improvement in survival rate of *Drosophila* by improving locomotor functions in the adult flies (134). Aging negatively influences the circadian clock heme oxygenase is one of the enzymes under control of the circadian clock in Drosophila and mammals as well. One mg/ml medium of curcumin was fed with standard diet elevated *ho* mRNA levels (135). *Caenorhabditis elegans* is a model nematode that is optimal for quick and efficient analysis of gene function because of its evident simplicity, precise genetics, availability of whole genome sequence, and full molecular toolset (142). Therefore, *C. elegans* has been used extensively to study curcumin's expertise in several parameters. Curcumin and along with other analogs, increased lifespan, improved locomotive activity, reduced fat accumulation, modulation of oxidative stress resistance, protection from microbes, and lowering manifestation of different types of diseases (143–146). Effect of curcumin on model insects and organisms are depicted in Table 4.

**Table 4:** Evaluation of curcumin's beneficial effects in the model insects and organisms.

**S. No**.	**Study**	**Effective dosage**	**Insect**	**Main findings**	**References**
1	Curcumin stimulates biochemical mechanisms of *Apis Mellifera* resistance and extends the apian life span	3 μg/ml	*Apis mellifera* (Western honey bee)	Curcumin increased apian life span, increased oxidation related protein levels and decreased DNA-methylation	(132)
2	Curcumin supplementation increases survival and lifespan in *Drosophila* under heat stress conditions	0.2 mg/g	*Drosophila melanogaster* (Fruit fly)	Curcumin fed drosophila were observed to have increased survival rates with enhance thermal tolerance	(133)
3	Developmental and behavioral toxicity induced by acrylamide exposure and amelioration using phytochemicals in *Drosophila melanogaster*	10 μM	*Drosophila melanogaster* (Fruit fly)	Curcumin along with another phytochemical have the potential in reducing acrylamide induced toxicity in *Drosophila melanogaster*	(134)
4	Regulation of heme oxygenase and its cross-talks with apoptosis and autophagy under different conditions in *Drosophila*	1 mg/mL (of the medium)	*Drosophila melanogaster* (Fruit fly)	Curcumin induced increased expression levels of heme oxygenase in drosophila brain	(135)
5	Screening bioactive food compounds in honey bees suggests curcumin blocks alcohol-induced damage to longevity and DNA methylation	100 μg/ml	*Apis mellifera* (Western honey bee)	Lifespan shortening caused by ethanol intake was restored when curcumin was co-administered through DNA methylation changes	(136)
6	Impacts of diverse natural products on honey bee viral loads and health	100 ppm	*Apis mellifera* (Western honey bee)	Curcumin fed bees showed low levels of *Gilliamella*, a honeybee bacterial symbiont	(137)

## 7. A brief account of the safe dosage and toxicity of curcumin

Determining the safe and effective dosage of curcumin is a tedious task in livestock and poultry animals as well as insects. Looking at the aforementioned studies, we saw that researchers given different dosages of curcumin as a feed additive and observed the livestock and poultry performances. According to the research studies mentioned in previous sections, the optimal dose in weaned piglets ranged from 300 to 400 mg/kg (71, 72, 80), whereas in growing pigs, the optimal dose was found to be 200 mg/kg (76, 79). Similarly, researchers found that 100–200 mg/kg of curcumin for lamb and 450–900 mg /day for sheep were the best doses (49, 74). In poultry, under oxidative stress, the dosage was recommended to be 50–200 mg/kg, irrespective of age. In the case of heat stress, 50–200 mg/kg of curcumin has been shown to be optimal in many studies (88, 91, 101). The recommended dosage of curcumin, while regulating intestinal microbiota or any other parasitic infection, was 200–400 mg/kg (47, 48). Meanwhile, 400–450 mg/kg of curcumin was found to be suitable for reducing mycotoxins like Aflatoxins (97, 106). Jin et al. (103) and Zhang et al. (94) suggested that 400–500 mg/kg of curcumin is best for improving meat quality in ducks, whereas 50–100 mg/kg of curcumin in broilers. In another study, 150 mg/kg of curcumin has a positive impact on increasing egg production (99). Curcumin's role in improving poultry and livestock production by enhancing their meat quality, egg-laying performance, and other productive traits has been demonstrated by various researchers. However, it should be noted that the beneficial dosage of curcumin still remains a debating issue. Since different studies have various experimental protocols and designs, it is difficult to conclude the beneficial and safe dose for practical usage. Standardizing the correct dosage regime of curcumin as feed additives for a particular function is the demand of the situation. Therefore, more research should be conducted in this direction. On the other hand, insect pest management studies conducted so far only examined curcumin's insecticidal activity; no studies investigated curcumin and their analogs side effects in plants, livestock, and poultry animals.

## 8. Conclusions and future prospects

Curcumin has been the subject of extensive investigation during the last few decades. Along with other biomedical applications, curcumin is an excellent nutraceutical candidate for animals and also a good insect repellent. Our aim with this review was to bring together the most recent available literature information and a thorough assessment based on the existing data. Livestock and poultry nutrition influence human health directly or indirectly since a significant part of the everyday meal comes from animals and their products. Insect vector-borne diseases and agricultural insect pests are getting increasingly worrisome, and the over use of chemical insecticides and unpredictable climatic changes are responsible for insecticide resistance in them. Therefore, it is necessary to control insect pests, especially using natural products such as curcumin, to avoid the hazardous effects of chemical insecticides. So, there will be a massive space of research opportunities in developing curcumin as a nutraceutical candidate and insect repellent.

Curcumin is proven to have many beneficial effects in maintaining livestock and poultry animal's health and performance. Also, it is evident that curcumin can be a viable alternative to antibiotics. However, we still do not fully understand which biochemical route or mechanism is targeted by curcumin. This uncertainty arises because there can be some other factors influencing the specific animal to show positive modifications regardless of the issue being studied. In this context, detailed studies needed to be employed on a molecular level after the administration of curcumin. Also, quantitative and qualitative analyses of curcumin in different tissues of animals are needed. Beneficial dose and toxicity in relation to practical applications are yet to be determined. Despite evidence that curcumin is a healthy feed additive to replace antibiotics for livestock and poultry, comparative research between curcumin antibiotics is still lacking. Taken together, we propose that large-scale evaluations are needed to suggest the precise and beneficial dose to livestock and poultry. Moreover, studying the impact of curcumin on digestion in livestock and poultry animals is an area worthy of future research. There are certain insect pests that disrupt the healthy wellbeing of livestock and poultry animals called ectoparasites. They cause major economic loss by interrupting the good productivity of animals, followed by many factors such as poor growth and reproductive performance. So, we put forward the idea of investigating more on these ectoparasites and whether curcumin and its modified formulations have specific effects on them. Regardless of the evidence that curcumin shows insecticidal activity in various insect vectors and agricultural pests through larvicidal, ovicidal, and other growth-inhibiting activities, the large-scale application of curcumin are still limited, largely because of a lack of a comprehensive understanding of their modes of action. No proper molecular authentication or conclusive structure-activity relationship on how curcumin causes these effects inside the insect's biological system. Hence, deciphering the genetic and molecular mechanism behind the interplay can open wide research opportunities in developing insecticides. As such, a better understanding of the effects of curcumin makeup on the host will enable us to fully utilize the curcumin substances for economically effective and sustainable insect pest management. Moreover, in agricultural pests, adequate toxicity evaluations of the crops are required.

Recent advances in genomics and bioinformatics have created several opportunities for investigating the physiological effect, toxicity, and protein composition of egg and poultry meat proteins and the safe use of curcumin as an animal feed additive. Also, researchers should prioritize expanding the application of genomics and bioinformatics tools to understand the curcumin interaction on the biological systems of livestock, poultry, and insects. It is a known fact that curcumin nanoformulations are more effective than free curcumin. However, almost no studies have been conducted using them to improve animal performance and control insects. To effectively promote curcumin nanoformulations as a feed additive or insect repellent, precise information regarding the beneficial dose and toxicity of the particular compounds against the host is required. Additionally, the comparison of information between the advantages of free curcumin and curcumin nanoformulations is also needed. To be approved for use in livestock and poultry, curcumin nanoformulations first undergo appropriate characterization and large-scale toxicity testing evaluation. Moreover, before proposing curcumin nanoformulations for practical applications, more detailed research should also concentrate on the long-term consequences of these formulations. Further, it is good to use it in curcumin alone or in combination with other synthetic or natural substances as a feed additive or insect repellent. So additional research should be conducted in this direction. The inclusion of curcumin supplements in livestock and poultry feed seems possible in the near future. Even though the use of curcumin to control insect pests is still in its infancy, encouraging results from recent studies are driving further investigations.

## Author contributions

AS, TM, and AK: conceptualization. AS: literature collection and writing—original draft preparation. AS, ES, and AK: writing—review and editing. KN, GG, AK, TM, SK, and MM: suggestions and comments. All authors contributed to the article and approved the submitted version.
